# Chemical Profiling of Kaliziri Injection and Quantification of Six Caffeoyl Quinic Acids in Beagle Plasma by LC-MS/MS

**DOI:** 10.3390/ph15060663

**Published:** 2022-05-25

**Authors:** Changhua Liu, Atikanmu Wahefu, Xueying Lu, Rahima Abdulla, Jun Dou, Haiqing Zhao, Haji Akber Aisa, Xuelei Xin, Yongqiang Liu

**Affiliations:** 1 State Key Laboratory Basis of Xinjiang Indigenous Medicinal Plants Resource Utilization, Key Laboratory of Plant Resources and Chemistry of Arid Zone, Xinjiang Technical Institute of Physics and Chemistry, Chinese Academy of Sciences, Urumqi 830011, China; wowlch22045@163.com (C.L.); atikanmu18@mails.ucas.ac.cn (A.W.); xueyinglu@ms.xjb.ac.cn (X.L.); rahima@ms.xjb.ac.cn (R.A.); doujun@ms.xjb.ac.cn (J.D.); haiqing_zhq@ms.xjb.ac.cn (H.Z.); haji@ms.xjb.ac.cn (H.A.A.); 2 University of Chinese Academy of Sciences, Beijing 100049, China

**Keywords:** Kaliziri injection, caffeoyl quinic acid derivatives, UHPLC-Q-Orbitrap-MS, beagle plasma, tandem mass spectrometry, quantitative analysis

## Abstract

Vitiligo is a stubborn multifactorial skin disease with a prevalence of approximately 1% in the global population. Kaliziri, the seeds of *Vernonia anthelmintica* (L.) Willd., is a well-known traditional Uyghur medicine for the treatment of vitiligo. Kaliziri injections is a Chinese-marketed treatment approved by the China Food and Drug Administration for the treatment of vitiligo. The significant effects of Kaliziri injection have been thoroughly studied. However, chemical components studies and plasma quantification studies are lacking for Kaliziri injection. Ultra-high-performance liquid chromatography coupled with hybrid quadrupole orbitrap mass spectrometry was employed to comprehensively characterize the caffeoyl quinic acid derivatives present in Kaliziri injection. Based on accurate mass measurements, key fragmental ions and comparisons with reference standards, 60 caffeoyl quinic acid derivatives were identified in Kaliziri injections, including caffeoyl quinic acids, coumaroyl caffeoyl quinic acids, dicaffeoyl quinic acids, feruloyl caffeoyl quinic acids, and dicaffeoyl quinic acid hexosides. Moreover, an HPLC-MS/MS method was developed and validated for the quantitative analysis of 5-caffeoyl quinic acid, 4-caffeoyl quinic acid, 1,3-dicaffeoyl quinic acid, 3,4-dicaffeoyl quinic acid, 3,5-dicaffeoyl quinic acid and 4,5-dicaffeoyl quinic acid in beagle plasma. The quantitative HPLC-MS/MS method was applied to quantify these six major caffeoyl quinic acids in beagle plasma after the subcutaneous administration of Kaliziri injection. All of the six analytes reached their peak plasma of concentrations within 30 min.

## 1. Introduction

Vitiligo, caused by the loss of the function of melanocytes and melanin in skin and hair, is an autoimmune disease characterized by the appearance of white spots, with an estimated prevalence of approximately 1% in the global population [1,2]. Clinical and epidemiological investigations have shown that vitiligo is a complex multifactorial disease [3].

Contemporary treatment strategies for vitiligo include phototherapy, local or systemic immunosuppressive agents, and surgical treatment [4]. However, these treatments can only prevent the progression of the disease and promote the re-coloring of depigmented areas, but cannot completely cure vitiligo. Therefore, vitiligo’s recurrence has grave impacts on the physical health, quality of life and social communication of these patients resulting in some psychological disorders, such as the development of an inferiority complex and social isolation [5,6].

The seeds of *Vernonia anthelmintica* (L.) Willd. (Quchong Banjiuju) are called Kaliziri in traditional Uyghur medicine, and have traditionally been used for the treatment of vitiligo [7]. In a prior phytochemical investigation, we reported the isolation and structure elucidation of sesquiterpene components in petroleum ether extracts of Kaliziri (though by NMR spectroscopy). In particular, the 21 newly identified vernodalidimers are rare elemanolide type dimers [8]. The mechanism of Kaliziri extract against vitiligo has been studied from enzymology, cell, and molecule aspects. The results show that Kaliziri extract can directly activate tyrosinase activity in B16 melanocytes and promote the melanin content and tyrosinase activity of B16 melanocytes; RT-PCR and western blot experiments further revealed that it can promote the upregulation of several genes and proteins closely associated with melanin synthesis in B16 melanoma cells, including MITF, TYR, TRP1, and TRP2. It was also found that it can stimulate melanin production in B16 cells by activating the MAPK and cAMP/PKA signaling pathway [7,9,10,11,12,13,14].

Kaliziri injection (KZI) is a Chinese-marketed treatment approved by the China Food and Drug Administration with an approval number of Z20063652. KZI is a preparation of aqueous Kaliziri extract and is used for the treatment of vitiligo. It is widely used in clinical combined treatment, and the effect is significant. Clinically, 308 nm excimer light combined with KZI is used to treat vitiligo. Compared with a control group, the effective rate of a treatment group was 26.8% higher, demonstrating a significant curative effect and high safety [15]. KZI combined with a vitiligo pill also had a significant effect on vitiligo. After 12 weeks of treatment, the effective rate of a control group was 41.2%, whereas that of a treatment group was 73.5% [16]. In addition, KZI can significantly inhibit the proliferation of T cells and B cells significantly in mice, which is related to the dosage of the injection. At the same time, KZI can activate tyrosinase activity in mice [17].

However, the chemical components of KZI have not yet been investigated. Additionally, the plasma quantification of key components has not been studied. Tandem mass spectrometry can powerfully characterize chemical components in herbs and plants [18,19,20,21,22]. Ultra-high-performance liquid chromatography coupled with quadrupole orbitrap mass spectrometry (UHPLC-Q-Orbitrap-MS) hybridizes the high mass resolution of orbitrap and the excellent selectivity of the quadrupole [23]. Herein, UHPLC-Q-Orbitrap-MS was employed to comprehensively identify caffeoyl quinic acid derivatives present in KZI. Moreover, an HPLC-MS/MS method was developed for the simultaneous quantitative analysis of six caffeoyl quinic acids in beagle plasma after the subcutaneous injection of Kaliziri.

## 2. Results and Discussion

### 2.1. The Identification of Caffeoyl Quinic Acid Derivatives in KZI by UHPLC-Q-Orbitrap-MS

**Figure 1:**
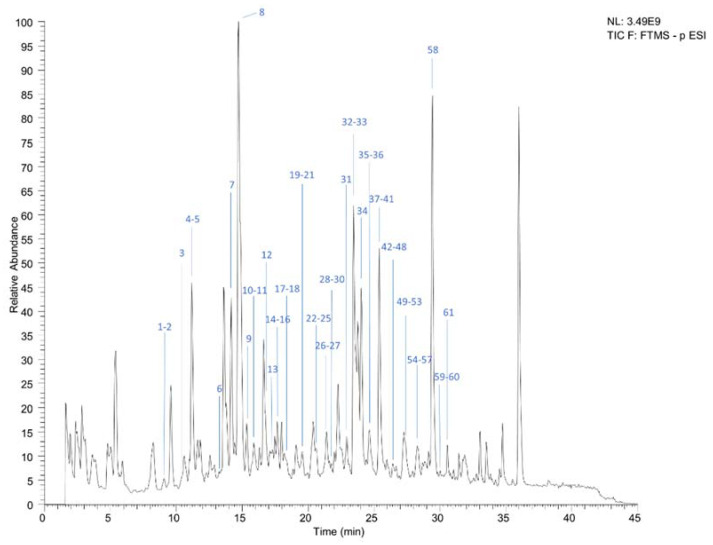
The total ion chromatogram of KZI by UHPLC-Q-Orbitrap-MS.

The UHPLC-Q-Orbitrap-MS total ion chromatogram (TIC) of KZI is shown in Figure 1. Based on the accurate mass measurements, the key fragmental ions and the comparison with reference standards, 60 caffeoyl quinic acid derivatives were identified in KZI. The results are shown in Table 1.

**Figure 2:**
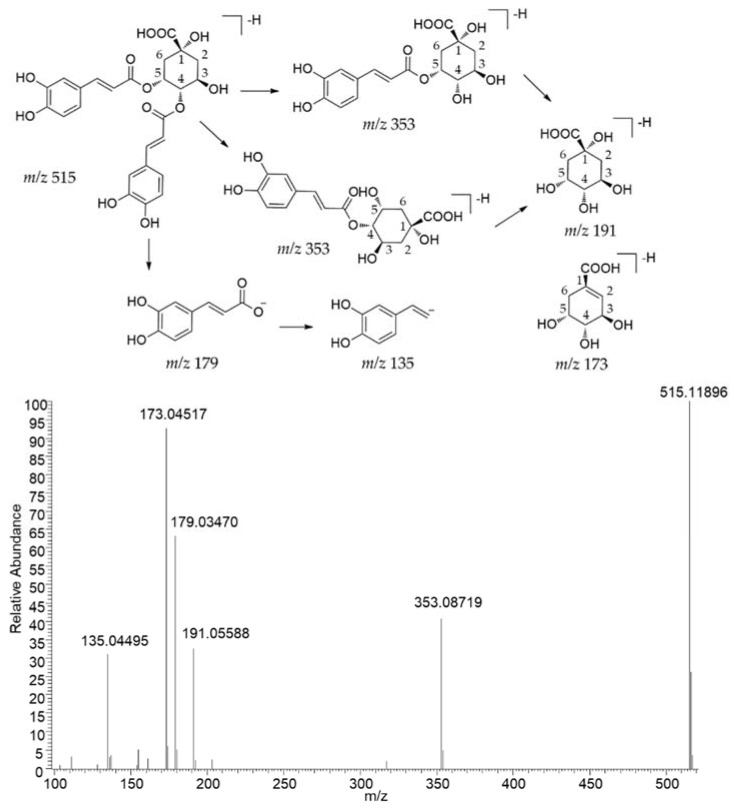
The fragment pathway of 4,5-dicaffeoyl quinic acid.

The typical fragmental ions of caffeoyl quinic acid derivatives were preliminarily determined by standards using UHPLC-Q-Orbitrap-MS. Typically, fragmental ions at *m*/*z* 191 (C_7_H_11_O_6_^−^) correspond to [quinic acid–H]^−^, *m*/*z* 173 (C_7_H_9_O_5_^−^) correspond to [quinic acid–H–H_2_O]^−^, and *m*/*z* 179 (C_9_H_7_O_4_^−^) corresponding to [caffeic acid–H]^−^. The fragment pathway of 4,5-dicaffeoyl quinic acid is shown in Figure 2.

Compounds **2**, **4**, **7,** and **8** exhibited the [M–H]^−^ ions at *m*/*z* 353, with a molecular formula of C_16_H_18_O_9_. In the MS/MS spectra, they revealed diagnostic [quinic acid–H]^−^ ions at *m*/*z* 191 [M–H–162]^−^ as well as a diagnostic loss of 162 Da (C_9_H_6_O_3_) for a caffeoyl moiety, suggesting that these compounds were caffeoyl quinic acids (CQAs). From comparisons of the retention time, high-resolution MS data, and MS/MS spectra data with those of authentic standards, compounds **4**, **7,** and **8** were identified as 3-caffeoyl quinic acid (3-CQA), 5-caffeoyl quinic acid (5-CQA) and 4-caffeoyl quinic acid (4-CQA). Compounds **6**, **12** and **13** gave [M–H]^−^ ions at *m*/*z* 337, with a molecular formula of C_16_H_18_O_8_. In the MS/MS spectra, their diagnostic [quinic acid–H]^−^ ion at *m*/*z* 191 [M–H–162]^−^ as well as diagnostic loss of 146 Da (C_9_H_6_O_2_) for a coumaroyl moiety suggesting that these compounds were coumaroyl quinic acids. Compounds **17** and **18** exhibited [M–H]^−^ ions at *m*/*z* 367, with molecular formula of C_17_H_20_O_9_. In the MS/MS spectra, their diagnostic [quinic acid–H]^−^ ion at *m*/*z* 191 [M–H–162]^−^ as well as the diagnostic loss of 176 Da (C_10_H_8_O_3_) for a feruloyl moiety suggested that compounds **17** and **18** were feruloyl quinic acids (FQAs). Compounds **10**, **14** and **16** exhibited the same [M-H]^−^ ions at *m*/*z* 399, with a molecular formula of C_18_H_24_O_10_. Their fragmental ions at *m*/*z* 353 ([CQA–H]^−^ and [M–C_2_H_6_O–H]^−^), as well as diagnostic ions at *m*/*z* 179 ([caffeic acid–H]^−^), indicated that these compounds were caffeoyl quinic acid ethyl esters.

**Figure 3:**
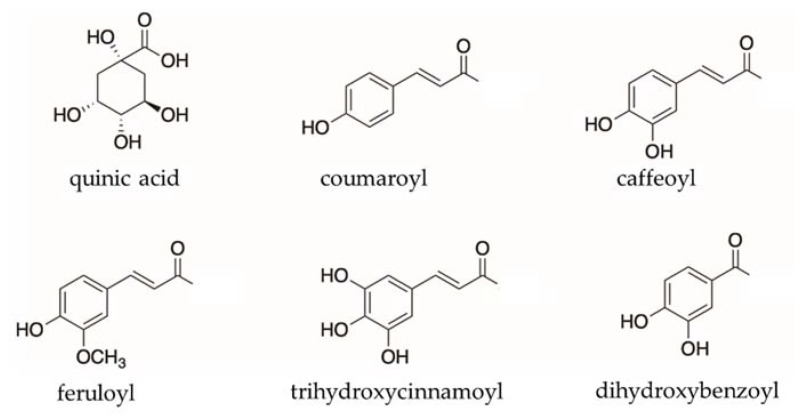
Selected structures of substituents associated with quinic acid.

Compounds **15**, **33**, **34** and **37** exhibited same [M–H]^−^ ions at *m*/*z* 515, with a molecular formula of C_25_H_24_O_12_. In the MS/MS spectra, they exhibited a diagnostic [CQA–H]^−^ ion at *m*/*z* 353 [M–H–162]^−^ and [quinic acid–H]^−^ ion at *m*/*z* 191 [M–H–162–162]^−^ suggesting that these compounds were dicaffeoyl quinic acids (diCQAs). From comparison of the retention time, high-resolution MS data, and MS/MS spectra data with those of authentic standards, compounds **15**, **33**, **34** and **37** were identified as 1,3-dicaffeoyl quinic acid (1,3-diCQA), 3,4-dicaffeoyl quinic acid (3,4-diCQA), 3,5-dicaffeoyl quinic acid (3,5-diCQA) and 4,5-dicaffeoyl quinic acid (4,5-diCQA), respectively. Compounds **22**, **27**, **40**, **41**, **43**, **45**, **53** and **54** exhibited the same [M–H]^−^ ions at *m*/*z* 499, with molecular a formula of C_25_H_24_O_11_. Their diagnostic fragmental ions at *m*/*z* 353 [CQA–H]^−^ or *m*/*z* 337 [CoQA–H]^−^, diagnostic ions at *m*/*z* 179 [caffeic acid–H]^−^ or *m*/*z* 163 [coumaric acid–H]^−^ and diagnostic ions at *m*/*z* 191 [quinic acid–H]^−^ indicated that these compounds were coumaroyl caffeoyl quinic acids (CoCQAs). Compounds **42**, **46**, **49**, **51**, **55** and **56** exhibited the same [M–H]^−^ ions at *m*/*z* 529, with a molecular formula of C_26_H_26_O_12_. Their diagnostic fragmental ions at *m*/*z* 353 [CQA–H]^−^ or *m*/*z* 367 [FQA–H]^−^, diagnostic ions at *m*/*z* 179 [caffeic acid–H]^−^ or *m*/*z* 193 [ferulic acid–H]^−^ and diagnostic ions at *m*/*z* 191 [quinic acid–H]^−^ indicated that these compounds were feruloyl caffeoyl quinic acids. Compounds **9**, **24**, **26** and **31** exhibited the same [M–H] ^−^ ions at *m*/*z* 489, with a molecular formula of C_23_H_23_O_12_. Their diagnostic fragmental ions at *m*/*z* 179 [caffeic acid–H]^−^ or *m*/*z* 153 [dihydroxybenzoic acid–H]^−^, diagnostic ions at *m*/*z* 353 [CQA–H]^−^ or *m*/*z* 327 [dihydroxybenzoyl quinic acid–H]^−^ and diagnostic ions at *m*/*z* 191 [quinic acid–H]^−^ indicated that these compounds were dihydroxybenzoyl caffeoyl quinic acids. Compounds **35**, **36**, **38**, **39**, **44**, **47**, **48**, **50** and **52** exhibited the same [M–H]^−^ ions at *m*/*z* 561, with a molecular formula of C_27_H_30_O_13_. In the MS/MS spectra, they exhibited diagnostic ions at *m*/*z* 515 [diCQA–H]^−^, *m*/*z* 353 [CQA–H]^−^, *m*/*z* 191 [quinic acid–H]^−^ and *m*/*z* 179 [caffeic acid–H]^−^, which indicated that they were derivatives of diCQA. Furthermore, the daughter ions at *m*/*z* 515 [M–C_2_H_6_O–H]^−^, *m*/*z* 399 [M–caffeoyl–H]^−^ and *m*/*z* 353 [M–caffeoyl–C_2_H_6_O–H]^−^ suggested that compounds **35**, **36**, **38**, **39**, **44**, **47**, **48**, **50** and **52** were dicaffeoyl quinic acid ethyl esters. The structures of substituents connect to quinic acid are shown in Figure 3.

Compound **57** exhibited [M–H]^−^ ions at *m*/*z* 677, with a molecular formula of C_34_H_30_O_15_. Its diagnostic fragmental ions at *m*/*z* 515 ([diCQA–H]^−^, [M–caffeic acid–H]^−^), *m*/*z* 353 [CQA–H]^−^, *m*/*z* 191 [quinic acid–H]^−^ and *m*/*z* 179 [caffeic acid–H]^−^ indicated that compound **57** was tricaffeoyl quinic acid. Compounds **10**, **19**, **20**, **21**, **23** and **25** exhibited the same [M–H]^−^ ions at *m*/*z* 693, with a molecular formula of C_34_H_30_O_16_, one oxygen more than compound **57**. In the MS/MS spectra, their diagnostic ions at *m*/*z* 353 [CQA–H]^−^, *m*/*z* 191 [quinic acid–H]^−^ and *m*/*z* 179 [caffeic acid–H]^−^ indicated that they were derivatives of CQA. Moreover, their fragmental ions at *m*/*z* 531 [M–caffeoyl–H]^−^ and *m*/*z* 353 [M–caffeoyl–trihydroxycinnamoyl–H]^−^ suggested that these compounds were trihydroxycinnamoyl-dicaffeoyl quinic acids.

Compounds **28**, **29**, **30** and **32** exhibited the same [M–H]^−^ ions at *m*/*z* 677.17371, with a molecular formula of C_31_H_34_O_17_. In the MS/MS spectra, their diagnostic ions at *m*/*z* 353 [CQA–H]^−^, *m*/*z* 191 [quinic acid–H]^−^ and *m*/*z* 179 [caffeic acid–H]^−^ indicated that they were derivatives of CQA. Meanwhile, the daughter ions at *m*/*z* 515 ([diCQA–H]^−^, [M–C_6_H_10_O_5_–H]^−^ ) indicated the existence of a hexosyl group and another caffeoyl group. Thus, compounds **28**, **29**, **30,** and **32** were identified as dicaffeoyl quinic acid hexosides. Compounds **1**, **3** and **5** exhibited the same [M–H]^−^ ions at *m*/*z* 677.19519, with a molecular formula of C_28_H_38_O_19_. In the MS/MS spectra, their diagnostic ions at *m*/*z* 353 [CQA–H]^−^, *m*/*z* 191 [quinic acid–H]^−^ and *m*/*z* 179 [caffeic acid–H]^−^ indicated they were derivatives of CQA. Moreover, their fragmental ions at *m*/*z* 515 [M–C_6_H_10_O_5–_H]^−^ and *m*/*z* 353 [M–C_6_H_10_O_5_–C_6_H_10_O_5–_H]^−^ indicated the existence of two hexosyl groups. Compounds **1**, **3**, and **5** were therefore identified as caffeoyl quinic acid hexosyl hexosides.

### 2.2. Simultaneous Quantification of 5-CQA, 4-CQA, 1,3-diCQA, 3,4-diCQA, 3,5-diCQA and 4,5-diCQA in Beagle Plasma after the Subcutaneous Injection of KZI

#### 2.2.1. Optimization of HPLC-MS/MS Conditions for Quantitative Analysis

To quantify these six major caffeoyl quinic acids in beagle plasma, an XDB-C18 column using the optimized mobile phase weas employed for sharp peak shapes with high intensity. The ion transition pairs were *m*/*z* value 353/191 for 5-CQA and 4-CQA; *m*/*z* value 515/353 for 1,3-diCQA, 3,4-diCQA, 3,5-diCQA and 4,5-diCQA; and *m*/*z* value 447/285 for astragalin, the internal standard. Optimized parameters are shown in Table 2. The major fragmentation of 5-CQA was the same as that of 4-CQA, and similar for 1,3-diCQA, 3,4-diCQA, 3,5-diCQA and 4,5-diCQA. In order to separate them in the shortest time with a satisfactory chromatographic resolution, the HPLC method was optimized (Section 3.3). The optimal retention times were 7.5, 8.7, 9.8, 14.0, 14.9, and 16.6 min for 5-CQA, 4-CQA, 1,3-diCQA, 3,4-diCQA, 3,5-diCQA and 4,5-diCQA, respectively.

#### 2.2.2. Method Validation

Specificity. Specificity was examined using blank beagle plasma and blank beagle plasma with added in 5-CQA, 4-CQA, 1,3-diCQA, 3,4-diCQA, 3,5-diCQA and 4,5-diCQA (each at 100.0 ng/mL) and the internal standard (100.0 ng/mL). No interference was found.

Calibration curves and linearity. Calibration curves were calculated using the peak area ratio of analytes compared with the internal standard. A weighting factor of 1/X^2^ was used for linearity. The method demonstrated strong linear reliability. The six analytes showed linearity with concentration ranging from 6 to 600 ng/mL in beagle plasma. The lower limit of quantification (LLOQ) for 5-CQA, 4-CQA, 1,3-diCQA, 3,4-diCQA, 3,5-diCQA and 4,5-diCQA was 6 ng/mL.

Accuracy and precision. The quality control (QC) sample solutions were prepared in beagle plasma for 5-CQA, 4-CQA, 1,3-diCQA, 3,4-diCQA, 3,5-diCQA and 4,5-diCQA, each at 10, 100, and 400 ng/mL. Intra-day accuracy and precision were evaluated in six replicates of QC samples with different concentrations on the same day. Inter-day accuracy and precision were assessed in triplicates of QC samples with different concentrations on three days. Accuracy was calculated by comparing the mean concentration to the theoretical concentration. Precision was interpreted by the relative standard deviation (RSD). The intra-day and inter-day accuracy of six analytes at each concentration were in the range of 85–115%. The intra-day and inter-day precision of six analytes at each concentration were in the RSD range of 0–15%.

Stability. Long-term stability was evaluated in three replicates at different QC concentrations after storage at −80 °C for 20 days. Freeze–thaw stability was assessed in three replicates of different QC concentrations after exposure to three sequential freeze-thaw cycles. For each cycle, samples were frozen for more than 24 h below −80 °C, then transferred to a 4 °C environment for 2 h until completely thawed. Stability in an auto-sampler was evaluated in three replicates at different QC concentrations. Six analytes at each concentration were stable over 12 h in the auto-sampler. They were also stable after three freeze-thaw cycles and after 20 days of storage at −80 °C.

#### 2.2.3. Simultaneous Quantification of 5-CQA, 4-CQA, 1,3-diCQA, 3,4-diCQA, 3,5-diCQA and 4,5-diCQA in Beagle Plasma after the Subcutaneous Injection of KZI

**Figure 4:**
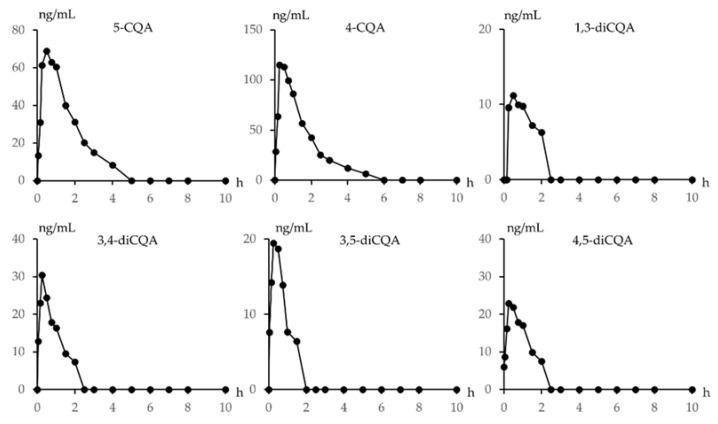
Concentration-time profiles of 5-CQA, 4-CQA, 1,3-diCQA, 3,4-diCQA, 3,5-diCQA, and 4,5-diCQA in beagle plasma after the subcutaneous injection of KZI.

The contents of 5-CQA, 4-CQA, 1,3-diCQA, 3,4-diCQA, 3,5-diCQA and 4,5-diCQA in KZI were determined as shown in Table 3. The quantitative HPLC-MS/MS method was applied to quantify 5-CQA, 4-CQA, 1,3-diCQA, 3,4-diCQA, 3,5-diCQA and 4,5-diCQA in beagle plasma samples. After the subcutaneous injection of KZI, beagle plasma exhibited quantifiable levels for all of the six analytes. As shown in Figure 4: At 0.5 h after the subcutaneous injection of KZI, 5-CQA reached the peak plasma concentration (61.23 ng/mL); 5 h after administration, the level of 5-CQA fell to under the LLOQ. At 0.25 h after the administration of KZI, 4-CQA reached the peak plasma concentration (114.87 ng/mL); 6 h after administration, the level of 4-CQA fell to under the LLOQ. At 0.5 h after administration of KZI, 1,3-diCQA reached the peak plasma concentration (11.20 ng/mL); 2.5 h after administration, the level of 1,3-diCQA fell to under the LLOQ. At 0.25 h after the administration of KZI, 3,4-diCQA reached the peak plasma concentration (30.46 ng/mL); 2.5 h after administration, the level of 3,4-diCQA fell to under the LLOQ. At 0.25 h after administration of KZI, 3,5-diCQA reached the peak plasma concentration (19.49 ng/mL); 2 h after administration, the level of 3,5-diCQA fell to under the LLOQ. At 0.25 h after administration of KZI, 4,5-diCQA reached the peak plasma concentration (22.96 ng/mL); 2.5 h after administration, the level of 4,5-diCQA fell to under the LLOQ.

## 3. Materials and Methods

### 3.1. Materials

Reference standards of astragalin were purchased from MUST-Biological (Chengdu, China). 5-caffeoyl quinic acid, 4-caffeoyl quinic acid, 1,3-di-caffeoyl quinic acid, 3,4-di-caffeoyl quinic acid, 3,5-dicaffeoyl quinic acid and 4,5-dicaffeoyl quinic acid were provided by Xinjiang Technical Institute of Physics and Chemistry (Urumqi, China). Kaliziri Injections (Batch No. 190407) were purchased from Wuhu Yangyan Pharmaceutical Co., Ltd., (Wuhu, China). MS-grade methanol, acetonitrile and formic acid were purchased from Merck (Darmstadt, Germany) and Fisher Scientific (Fair Lawn, NJ, USA).

### 3.2. UHPLC-Q-Orbitrap-MS Conditions

Qualitative analysis was performed on Q Exactive Orbitrap apparatus coupled with Ultimate 3000 equipment (Thermo Fisher Scientific, Waltham, MA, USA). The column was an HSS T_3_ (1.8 μm, 2.1 × 100 mm, Waters, Ireland). The column oven temperature was 40 °C. The mobile solvents were A (acetonitrile, 0.1% *v*/*v* formic acid) and B (water, 0.1% *v*/*v* formic acid) a with flow rate of 250 μL/min and in the following gradients: 0.0–2.0 min (0% A), 2.0–25.0 min (0–23% A), 25.0–40.0 min (23–83% A), 40.0–40.1 min (83–100% A), 40.1–45.0 min (100% A). The injected volume was 1 μL. Electrospray ionization (ESI) was employed in positive mode and negative mode. The *m*/*z* range was 100–1200 with a resolution of 70,000. Voltage: 3.8 kV; sheath gas flow: 40 arb; auxiliary gas flow: 10 arb; heating temperature: 350 °C; capillary temperature: 350 °C; stepped normalized collision energy: 15, 40 and 65. The data were processed using the Xcalibur 4.2 software (Thermo Fisher Scientific, Waltham, MA, USA).

### 3.3. Quantitative HPLC-MS/MS Conditions

Quantitative HPLC-MS/MS analyses were performed using a 1200 system coupled with 4000 Q TRAP (Applied Biosystems/MDS Sciex, Framingham, MA, USA). The column was XDB-C18 (1.8 μm, 4.6 × 50 mm, Agilent, Santa Clara, CA, USA). The mobile solvents were A (acetonitrile, 0.2% *v*/*v* formic acid) and B (water, 0.2% *v*/*v* formic acid) with a flow rate of 0.6 mL/min and in the following gradients: 0.0–6.0 min (10–12% A), 6.0–6.1 min (12–20% A), 6.1–19.0 min (20–26% A). The injection volume was 10 μL. The MS was in ESI-negative mode with multiple reaction monitoring (MRM). IonSpray voltage: −4000 V; nebulizer gas: 45; auxiliary heater gas: 55; curtain gas: 30; turbo gas temperature: 500 °C. The data were acquired with the Analyst 1.6.2 software (Applied Biosystems/MDS Sciex, Framingham, MA, USA) and processed with the MultiQuant 2.1 software (Applied Biosystems/MDS Sciex, Framingham, MA, USA).

### 3.4. Animal Experiment

Three male beagles were obtained from Xinjiang Medical University. They were fasted for 12 h before administration, but drank water freely. Animals were administrated subcutaneous KZI injection with a dose of 0.2 mL/kg. Blood was collected at 0, 0.05, 0.15, 0.25, 0.5, 0.75, 1, 1.5, 2, 2.5, 3, 4, 5, 6, 7, 8, and 10 h from hind limbs veins. Urine and feces were also collected. The collected blood was centrifuged for 15 min to produce plasma samples. The plasma samples were immediately stored at −80 °C.

### 3.5. Beagle Plasma Standard Solution Preparation

The stock solutions of 5-CQA, 4-CQA, 1,3-diCQA, 3,4-diCQA, 3,5-diCQA, 4,5-diCQA and the internal standard, astragalin, were prepared by dissolving each compound in methanol to concentrations of 2.0 mg/mL and stored at −20 °C. Calibration curve standard sample preparation was as follows: 10 μL appropriately diluted standard solutions (mixtures of 5-CQA, 4-CQA, 1,3-diCQA, 3,4-diCQA, 3,5-diCQA and 4,5-diCQA) was added into blank beagle plasma and vortexed for 10 s to result in samples containing 6–600 ng/mL standard compounds. The quality control (QC) sample solutions were prepared in beagle plasma for 5-CQA, 4-CQA, 1,3-diCQA, 3,4-diCQA, 3,5-diCQA and 4,5-diCQA, each at 10, 100, and 400 ng/mL.

### 3.6. Treatment of Beagle Plasma Samples

Beagle plasma samples were mixed with 10 μL internal standard solution and vortexed for 10 s. Subsequently, 300 μL acetonitrile was added and the mixture was vortexed for 60 s; then, it was centrifuged for 7 min. The supernatant was collected and nitrogen dried at 40 °C. The dried samples were mixed with 100 μL methanol and sonicated for 60 s, then centrifuged for 7 min. The supernatant was collected and injected for analysis.

## 4. Conclusions

In this study, UHPLC-Q-Orbitrap-MS was employed to comprehensively characterize the caffeoyl quinic acid derivatives present in KZI. Based on accurate mass measurements, key fragmental ions and comparison with reference standards, 60 caffeoyl quinic acid derivatives were identified in KZI, including caffeoyl quinic acids, coumaroyl caffeoyl quinic acids, dicaffeoyl quinic acids, feruloyl caffeoyl quinic acids, dicaffeoyl quinic acid hexosides, etc. Moreover, an HPLC-MS/MS method was developed and validated for the quantitative analysis of 5-CQA, 4-CQA, 1,3-diCQA, 3,4-diCQA, 3,5-diCQA and 4,5-diCQA in beagle plasma. The quantitative HPLC-MS/MS method was applied to quantify these six major caffeoyl quinic acids in beagle plasma after the subcutaneous injection of KZI. All of the six analytes reached their peak plasma concentration within 0.5 h. The 5-CQA and 4-CQA samples were quantifiable until a time point of 5 h. However, 1,3-diCQA, 3,4-diCQA, 3,5-diCQA and 4,5-diCQA fell under there LLOQs within 2.5 h.

## Figures and Tables

**Table 1:** Caffeoyl quinic acid derivative characterization of KZI by UHPLC-Q-Orbitrap-MS.

No.	*t*_R_ (min)	Molecular Formula	[M-H]^−^	Major and Important MS^2^ Ions	Identification	Error (ppm)
1	9.22	C_28_H_38_O_19_	677.19519	515, 353, 341, 191, 179, 173, 161, 135	CQA hexosyl hexoside-a	4.19
2	9.32	C_16_H_18_O_9_	353.08719	191	CQA	1.36
3	10.13	C_28_H_38_O_19_	677.19562	515, 353, 323, 191, 179, 173, 161, 135	CQA hexosyl hexoside-b	4.82
4	11.18	C_16_H_18_O_9_	353.08566	191, 179, 135	3-CQA *	−2.90
5	11.51	C_28_H_38_O_19_	677.19537	515, 353, 341, 179, 173, 135	CQA hexosyl hexoside-c	4.46
6	13.28	C_16_H_18_O_8_	337.09326	191, 173, 163, 119	CoQA-a	4.35
7	14.15	C_16_H_18_O_9_	353.08627	191	5-CQA *	−1.20
8	14.86	C_16_H_18_O_9_	353.08517	191, 179, 173, 135	4-CQA *	−4.30
9	15.47	C_23_H_22_O_12_	489.10510	353, 335, 191, 179, 161, 135, 109	dihydroxybenzoyl CQA-a	4.80
10	15.81	C_18_H_24_O_10_	399.13062	353, 191, 179, 135	CQA ethyl ester-a	5.12
11	16.07	C_34_H_30_O_16_	693.14832	531, 353, 339, 313, 295, 269, 229, 191, 173, 159, 109	Trihydroxycinnamoyl diCQA-a	4.77
12	16.79	C_16_H_18_O_8_	337.09351	191, 173, 163, 119	CoQA-b	5.07
13	17.11	C_16_H_18_O_8_	337.09311	191, 173, 163, 137, 119	CoQA-c	3.90
14	17.54	C_18_H_24_O_10_	399.13055	353, 191, 179, 135	CQA ester-b	4.96
15	17.63	C_25_H_24_O_12_	515.12024	353, 191, 179, 135	1,3-diCQA *	3.56
16	17.73	C_18_H_24_O_10_	399.13043	353, 191, 179, 135	CQA ethyl ester-c	4.66
17	18.32	C_17_H_20_O_9_	367.10413	191, 134	FQA-a	4.81
18	18.42	C_17_H_20_O_9_	367.10413	193, 191, 173, 155, 134	FQA-b	4.81
19	19.19	C_34_H_30_O_16_	693.14783	531, 353, 339, 313, 295, 269, 229, 191, 179, 173, 159, 109	Trihydroxycinnamoyl diCQA-b	4.06
20	19.33	C_34_H_30_O_16_	693.14789	531, 353, 339, 313, 295, 269, 229, 191, 179, 173, 159, 109	Trihydroxycinnamoyl diCQA-c	4.15
21	19.52	C_34_H_30_O_16_	693.14801	531, 353, 339, 295, 269, 229, 191, 179, 173, 159, 135, 109	Trihydroxycinnamoyl diCQA-d	4.33
22	20.39	C_25_H_24_O_11_	499.12573	353, 335, 191, 179, 161, 135	CoCQA-a	4.50
23	20.42	C_34_H_30_O_16_	693.14764	531, 353, 339, 295, 267, 229, 191, 179, 173, 159, 135, 109	Trihydroxycinnamoyl diCQA-e	3.80
24	20.55	C_23_H_22_O_12_	489.10468	353, 335, 327, 309, 191, 179, 173, 161, 153, 135, 109	dihydroxybenzoyl CQA-b	3.93
25	20.61	C_34_H_30_O_16_	693.14752	531, 353, 339, 313, 295, 269, 229, 191, 179, 173, 159, 109	Trihydroxycinnamoyl diCQA-f	3.62
26	21.05	C_23_H_22_O_12_	489.10498	327, 191, 179, 153, 109	dihydroxybenzoyl CQA-c	4.56
27	21.11	C_25_H_24_O_11_	499.12570	337, 191, 163, 119	CoCQA-b	4.44
28	21.43	C_31_H_34_O_17_	677.17371	515, 353, 323, 191, 179, 173, 161, 135	diCQA hexoside-a	3.66
29	21.79	C_31_H_34_O_17_	677.17450	515, 353, 323, 191, 179, 173, 161, 135	diCQA hexoside-b	4.83
30	22.26	C_31_H_34_O_17_	677.17383	515, 353, 323, 191, 179, 173, 161, 135	diCQA hexoside-c	3.84
31	22.85	C_23_H_22_O_12_	489.10477	327, 191, 173, 153, 109	dihydroxybenzoyl CQA-d	4.12
32	23.31	C_31_H_34_O_17_	677.17444	515, 353, 323, 191, 179, 173, 161, 135	diCQA hexoside-d	4.75
33	23.50	C_25_H_24_O_12_	515.11975	353, 335, 191, 179, 173, 135	3,4-diCQA *	2.62
34	24.07	C_25_H_24_O_12_	515.12006	353, 191, 179, 135	3,5-diCQA *	3.21
35	24.76	C_27_H_30_O_13_	561.16260	515, 399, 353, 191, 179, 173, 161, 135	diCQA ethyl ester-a	4.15
36	24.99	C_27_H_30_O_13_	561.16266	515, 399, 353, 191, 179, 173, 161, 135	diCQA ethyl ester-b	4.26
37	25.42	C_25_H_24_O_12_	515.11987	353, 191, 179, 173, 135	4,5-diCQA *	2.85
38	25.51	C_27_H_30_O_13_	561.16193	515, 399, 353, 335, 191, 179, 173, 161, 135	diCQA ethyl ester-c	2.96
39	25.74	C_27_H_30_O_13_	561.16241	515, 399, 353, 335, 191, 179, 173, 161, 135	diCQA ethyl ester-d	3.83
40	25.77	C_25_H_24_O_11_	499.12561	353, 337, 335, 319, 191, 179, 173, 163, 135, 119	CoCQA-c	4.25
41	26.14	C_25_H_24_O_11_	499.12567	353, 337, 319, 191, 179, 173, 163, 119	CoCQA-d	4.37
42	26.31	C_26_H_26_O_12_	529.13617	365, 335, 193, 191, 179, 175, 173, 161, 135, 134	FCQA-a	4.00
43	26.41	C_25_H_24_O_11_	499.12570	337, 191, 173, 163, 119	CoCQA-e	4.44
44	26.41	C_27_H_30_O_13_	561.16266	499, 414, 399, 353, 191, 179, 173, 161, 135	diCQA ethyl ester-e	4.26
45	26.64	C_25_H_24_O_11_	499.12564	353, 337, 191, 179, 173, 135	CoCQA-f	4.31
46	26.81	C_26_H_26_O_12_	529.13617	367, 335, 193, 173, 161, 134	FCQA-b	4.00
47	26.85	C_27_H_30_O_13_	561.16272	515, 441, 399, 353, 191, 179, 173, 135	diCQA ethyl ester-f	4.37
48	27.28	C_27_H_30_O_13_	561.16254	515, 399, 353, 191, 179, 173, 135	diCQA ethyl ester-g	4.04
49	27.41	C_26_H_26_O_12_	529.13599	367, 193, 179, 134	FCQA-c	3.65
50	27.47	C_27_H_30_O_13_	561.16260	515, 399, 353, 191, 179, 173, 161, 135	diCQA ethyl ester-h	4.15
51	27.61	C_26_H_26_O_12_	529.13629	367, 353, 191, 179, 135	FCQA-d	4.23
52	27.61	C_27_H_30_O_13_	561.16278	515, 399, 353, 351, 191, 179, 173, 135	diCQA ethyl ester-i	4.48
53	27.81	C_25_H_24_O_11_	499.12567	337, 191, 173, 163, 119	CoCQA-g	4.37
54	27.98	C_25_H_24_O_11_	499.12576	353, 337, 191, 179, 173, 163, 135	CoCQA-h	4.56
55	28.19	C_26_H_26_O_12_	529.13593	367, 183, 173, 134	FCQA-e	3.54
56	28.38	C_26_H_26_O_12_	529.13562	367, 353, 335, 191, 179, 173, 135	FCQA-f	2.96
57	29.52	C_34_H_30_O_15_	677.15283	515, 353, 335, 191, 179, 173, 161, 135	triCQA	4.04
58	30.02	C_35_H_34_O_15_	693.18433	531, 513, 353, 335, 191, 179, 177, 173, 161, 135, 133	Hydroferuoyl diCQA	4.23
59	30.19	C_35_H_34_O_15_	693.18445	531, 335, 191, 179, 177, 173, 161, 135, 133	Hydroferuoyl diCQA	4.40
60	30.58	C_35_H_34_O_15_	693.18408	531, 353, 191, 179, 173, 135	Hydroferuoyl diCQA	3.87

* Identified by comparing with reference standard: CQA, caffeoyl quinic acid; CoQA, coumaroyl quinic acid; diCQA, dicaffeoyl quinic acid; FQA, feruloyl quinic acid; FCQA, feruloyl caffeoyl quinic acid; CoCQA, coumaroyl caffeoyl quinic acid; triCQA, tricaffeoyl quinic acid.

**Table 2:** Optimized mass spectrometry conditions for 5-CQA, 4-CQA, 1,3-diCQA, 3,4-diCQA, 3,5-diCQA, and 4,5-diCQA and the internal standard, astragalin.

Analytes	Q1 Mass (Da)	Q3 Mass (Da)	DP (Volts)	EP (Volts)	CE (Volts)	CXP (Volts)
5-CQA	353	191	65	10	30	15
4-CQA	353	191	65	10	30	15
1,3-diCQA	515	353	85	10	27	15
3,4-diCQA	515	353	85	10	27	15
3,5-diCQA	515	353	85	10	27	15
4,5-diCQA	515	353	85	10	27	15
Internal standard	447	285	100	10	36	10

**Table 3:** Contents of 5-CQA, 4-CQA, 1,3-diCQA, 3,4-diCQA, 3,5-diCQA, and 4,5-diCQA in KZI.

Analytes	5-CQA	4-CQA	1,3-diCQA	3,4-diCQA	3,5-diCQA	4,5-diCQA
Contents (μg/mL)	46.2	48.0	9.7	190.0	11.7	17.2

## Data Availability

Data sharing not applicable.
